# Effects of genetic and environmental factors on variations of seed heteromorphism in *Suaeda aralocaspica*

**DOI:** 10.1093/aobpla/plaa044

**Published:** 2020-08-24

**Authors:** Jing Cao, Ling Chen, Juan Wang, Jiajia Xing, Xiuyun Lv, Tayier Maimaitijiang, Haiyan Lan

**Affiliations:** 1 Xinjiang Key Laboratory of Biological Resources and Genetic Engineering, College of Life Science and Technology, Xinjiang University, Urumqi, China; 2 Institute of Horticulture Crops, Xinjiang Academy of Agricultural Sciences, Urumqi, China

**Keywords:** Germination, heterospermy, internal and external factor, seed ontogeny, *Suaeda aralocaspica*

## Abstract

Seed heteromorphism is an adaptive strategy towards adversity in many halophytes. However, the underlying mechanisms and ecological significance of seed heteromorphism have not been deeply explored. Using *Suaeda aralocaspica*, a typical C_4_ annual halophyte without Kranz anatomy, we studied seed morphology, differentiation of morphs and fruit-setting patterns, and correlated these traits with germination responses, seed characteristics and heteromorphic seed ratio. To elucidate the genetic basis of seed heteromorphism, we analysed correlated patterns of gene expression for seed development-related genes as well. We observed that *S. aralocaspica* produced three types of seed morph: brown, large black and small black with differences in colour, size, mass and germination behaviour; the latter two were further distinguished by their origin in female or bisexual flowers, respectively. Further analysis revealed that seed heteromorphism was associated with genetic aspects including seed positioning, seed coat differentiation and seed developmental gene expression, while variations in seed heteromorphism may be associated with environmental conditions, e.g. annual precipitation, temperature, daylight and their monthly distribution in different calendar years. Seed heteromorphism and its variations in *S. aralocaspica* show multilevel regulation of the bet-hedging strategy that influences phenotypic plasticity, which is a consequence of internal genetic and external environmental factor interaction. Our findings contribute to the understanding of seed heteromorphism as a potential adaptive trait of desert plant species.

## Teaser

Seed heteromorphism provides plants with alternative strategies for survival in unfavourable environments. Cao *et al.* classified a third type of seed morph in *Suaeda aralocaspica* besides the previously reported dimorphs (brown and black seeds), resulting in brown, large black and small black seeds; the latter two were distinguished by their origin in female or bisexual flowers, respectively. Meanwhile, they also revealed that the formation and variations of seed heteromorphism in *S. aralocaspica* are caused by the combination of genetic aspects (seed positioning, seed coat differentiation, gene expression, etc.) and environmental factors (annual precipitation, temperature, illumination, etc.).

## Introduction

Plants have evolved various strategies to cope with heterogeneous habitats in the whole life cycle (Venable and Lawlor 1980; Venable 1985), including variations in morphology, structure, physiology and molecular biology. Seed heteromorphism is a phenomenon in which an individual plant produces two or more distinct types of seeds, has commonly been observed in semi-arid, saline and other harsh environments (Imbert 2002) and may influence plant development at different stages (Mandák 1997; Imbert 2002). As seed dimorphism or polymorphism is important for understanding plant adaptability, much attention has been paid to this issue in different plant species, e.g. species of *Suaeda* (Cao *et al.* 2012; Li and Li 2015; Yang *et al.* 2015a), *Triticum* (Volis 2016), *Diptychocarpus* (Lu *et al.* 2010) and *Chenopodium* (Yao *et al.* 2010). So far, differences in colour, shape, size, mechanisms of dispersal and dormancy have been documented between heteromorphic seeds (Baskin *et al.* 2014).

It has become evident that many maternal effects have naturally been selected to act as a mechanism for adapting the phenotype in response to environmental heterogeneity (Mousseau and Fox 1998). Based on the documented outcomes, there are four types of maternal effects: anticipatory, selfish, bet-hedging and transmissive (Marshall and Uller 2007). Seed heteromorphism has been considered as a bet-hedging strategy in adaptation to unpredictable environments in some annual plants (Wang *et al.* 2008; Lenser *et al.* 2016). Meanwhile, differences in seed size, germination behaviour and salt tolerance of the offspring seed due to the impacts of the salinity of the maternal environment and nutrient limitation are anticipatory maternal effects (Wang *et al.* 2012a), e.g. *Senecio vulgaris* grown at a higher nutrient level produces seeds that germinate earlier than those from plants grown at a lower nutrient level (Aarssen and Burton 1990). The phenotypic plasticity of some heteromorphic species is regarded as a mechanism for dealing with unpredictable environments (Molina-Montenegro *et al.* 2010; Shemesh and Novoplansky 2013; Furness *et al.* 2015). Several studies have demonstrated that, in some heteromorphic plant species, fruit and seed morph ratios and numbers (Lenser *et al.* 2016), dispersal ability (Imbert and Ronce 2001), plant size (Talavera *et al.* 2010) and biomass (Yang *et al.* 2015b) showed plasticity in response to certain environmental stimuli. Other environmental changes, e.g. precipitation, may also contribute to variations in seed heteromorphism (Li *et al.* 2005; Volis 2016).

Seed heteromorphism is often associated with different seed colours (Brändel 2004; Yao *et al.* 2010), which is determined by genetic background combined with a substantial environmental influence (Graeber *et al.* 2012). Seed coloration is complex due to the involvement of various pigments including flavonols, proanthocyanidin and maybe some other phenolic relatives, like lignin (Kleindt *et al.* 2010; Gu *et al.* 2011; Wan *et al.* 2016). Several lines of evidence have shown that *TRANSPARENT TESTA* (*TT*) types of genes and their homologues in *Arabidopsis* are involved in the accumulation of flavonoids or phenylpropanoids during the coloration of seeds (Yu 2013; MacGregor *et al.* 2015). *Arabidopsis BANYULS* (*BAN*) (anthocyanidin reductase, also called *ANR*) and *ANTHICYANIDIN SYNTHASE* (*ANS*) are also key enzymes in pigment biosynthesis, during which the seed coat colour is formed (Nesi *et al.* 2009; MacGregor *et al.* 2015). In many cases, seeds with different colours are associated with variation in seed dormancy (Imbert 2002; Torada and Amano 2002; Childs *et al.* 2010). The accumulation of transcription factor (TF) bHLH92 in *Leymus chinensis* can inhibit the expression of *BAN* and *ANS* genes in seeds, which results in a decrease in anthocyanins/proanthocyanidin biosynthesis and produces yellow seeds with weak dormancy (Zhao *et al.* 2019). Different genes related to seed dormancy have been characterized (Graeber *et al.* 2012). Four TFs, ABSCISIC ACID-INSENSITIVE 3 (ABI3), FUSCA 3 (FUS3), LEAFY COTYLEDON 1 (LEC1) and LEC2 are known to play a central role in the regulation of seed maturation and dormancy in *Arabidopsis* (Baumbusch *et al.* 2004); several other factors, e.g. maize *VIVIPAROUS 8* (*VP8*), have been shown to modulate the above four TFs, and a mutation of *VP8* gene causes a viviparous seed phenotype with pleiotropic developmental changes (Suzuki *et al.* 2008). Other genes, e.g. *DELAY OF GERMINATION 1* (*DOG1*), *EARLY FLOWERING IN SHORT DAYS* (*EFS*) in *Arabidopsis* and *SEED DORMANCY 4* (*SDR4*) in rice, also help control seed dormancy (Bentsink *et al.* 2006; Sugimoto *et al.* 2010; Bassel *et al.* 2011).

*Suaeda aralocaspica* (Amaranthaceae) is an annual halophyte with succulent leaves that grows in saline–alkaline sandy soil. In China, it is distributed in the inland cold desert of the Junggar Basin in Xinjiang province (Commissione Redactorum Florae Xinjiangensis 1994). Previous reports demonstrated that *S. aralocaspica* has a unisexual flower (monoecious plant) (Commissione Redactorum Florae Xinjiangensis 1994) and can produce two distinct seed morphs (brown and black), which have different dormancy characteristics and salt tolerance (Wang *et al.* 2008). Further studies suggested that seed heteromorphism, nutrient and salinity interact with each other in determining a range of seed traits of *S. aralocaspica* via bet-hedging and anticipatory maternal effects (Wang *et al.* 2012a). However, little is known about the effects of the genetic and environmental factors or their interaction on seed heteromorphism and its variations. Therefore, in the present study, we performed a series of experiments to address the following aspects: (i) to identify and characterize a third type of seed morph in *S. aralocaspica*; (ii) to elucidate the ontogeny of heteromorphic seeds in *S. aralocaspica*; (iii) to explore the internal (or genetic) causes (e.g. seed-setting pattern, cytology of seed coat development, expression profile of seed development-related genes, etc.) of seed heteromorphism in *S. aralocaspica*; (iv) to analyse the effect of external (or environmental) factors (e.g. precipitation, temperature, daylight, etc.) on variations in the seed morph ratio. Based on these, we expected to achieve an insight into the mechanism and significance of the formation and variation of seed heteromorphism in *S. aralocaspica*.

## Methods

### Seed collection

Mature seeds of *S. aralocaspica* were collected from dry inflorescences in natural populations growing in saline desert soil **[see**  **Supporting Information—Table S1****]** in Wujiaqu 103 regiment (44°37′N, 87°26′E; 423 mH), Xinjiang, China, in October from 2008 to 2012 calendar years. For the preparation of clean seeds, the above-ground parts of plants were carefully harvested to avoid shaking off the dry fruits, which were then air-dried indoors with stirring every day until completely dry (after 3–4 weeks), then the fruits were rubbed properly to release the intact seeds, which were then separated from the impurities and finally stored at room temperature or 4 °C in brown paper bags for later use.

### Meteorological data collection

Meteorological data of the natural habitat of *S. aralocaspica* during the previous 5 years (2008–12) were analysed by Xinjiang Meteorological Information Center (China), including monthly average temperature, monthly and annual precipitation and hours of daylight.

### Seed morphology observation

Thousands of intact mature seeds of *S. aralocaspica* were mixed well and randomly divided into several equal parts by weighing them, and one part was taken for measurement. The brown seeds were first removed from the mixture, then the remaining black seeds were separated into the smallest seeds (small black) and the other black seeds (large black). A stereomicroscope SMZ800 (Nikon, Japan) was employed to examine the seed surface with or without the seed coat. The diameter and thickness of different types of seeds were measured by vernier calliper (four replicates with 30 seeds of each seed type); the mass of 1000 seeds was weighed using an electronic analytical balance (four replicates with 1000 seeds in each). Digital photographs were manipulated with Adobe Photoshop (Adobe Photosystems) to prepare figures.

### Observation of the cytology during seed development

Paraffin sections were prepared to visualize the differentiation of heteromorphic seeds. When the bisexual flowers bloomed, the female flowers (developing fruits) at different positions on the inflorescence were fixed every 3 days for a total of 20 days. For fixation, the fruits were vacuum-infiltrated in FAA solution [50 % (the earlier stage) or 70 % (the later stage) ethanol:formalin:glacial acetic acid = 8:1:1] and left overnight at 4 °C. Then, tissues were dehydrated in 50 % ethanol (30 min, twice) followed by 1 % Safranine-O solution (in 70 % ethanol) staining for 8–10 h at room temperature, then dehydrated in an ethanol series from 80 % to absolute ethanol. For clearing, the dehydrated tissues were treated with xylene and absolute ethanol, then fine first-grade paraffin powder was added for solidification and the tissues were placed in an oven at 38 °C overnight. For paraffin inclusion, the above tissues were incubated at 56 °C for 1 h until the wax was completely melted, then transferred into melted second-grade paraffin at 57 °C for 1 h and finally submerged in third-grade paraffin at 58 °C for 40 min and 59 °C for 30 min (two changes). For paraffin embedding, the melted paraffin was poured into a paper tank and the tissue was adjusted into the proper position, then the paper tank was quickly placed into iced water to solidify the paraffin without generating bubbles. For sectioning, cutting and trimming the paraffin block (with the tissue in the middle) into a small cuboid to fit onto the paraffin loading stick, 8–10 μm sections were prepared with a Leica RM 2126 microtome. To expand the section, the paraffin sections were retrieved and spread on a glass slide with egg white as the adhesive agent, and then placed at 37 °C in an oven overnight. For deparaffinization, the glass slide with sections was successively incubated in xylene, xylene + ethanol and ethanol. For Safranine-Fast green counterstaining, the slides were transferred to 80 % ethanol for 3–5 min, followed by 1 % Safranine-O solution (prepared in 70 % ethanol) staining for 8–10 h at room temperature; after treatment with 80 % ethanol for another 3–5 min, the slide was quickly and gently dipped into 1 % Fast green solution (prepared in 95 % ethanol) for ~10 s, and immediately transferred into a series of ethanol, ethanol + xylene and xylene. For mounting, one drop of neutral gum was quickly applied over the specimen and a cover slip was added and sealed with nail polish and then incubated at 37 °C for drying. For microscopy examination, a Leica DM3000 light microscope (Leica Microsystems, Germany) was used; photographs were taken using the LAS V4.0 program.

### Investigation of fruit-setting pattern in *S. aralocaspica*

Dimorphic seeds of *S. aralocaspica* were sown in bamboo baskets (50 cm × 40 cm × 30 cm in length × width × height) containing saline–alkaline soil (collected from the natural habitat) and kept outdoors in a garden, which was surrounded by buildings on three sides, and a fence on the fourth side. The garden was exposed to natural sunlight, precipitation and free air. Seed sowing was carried in two seasons to simulate natural conditions: first in deep Autumn when the soil surface was close to 0 °C and second in the following Spring after snow melting. Plants were cultivated for 4–5 months until flowering and seed production. In the growth period, plants were supplied with sufficient water at an interval of 1 week in Spring or 3–4 days in Summer. The fruit-setting pattern of surviving plants was recorded. During the flowering initiation stage (August), female or bisexual flowers were sampled from different positions on the inflorescence every 2 days and examined by stereomicroscope. In the fruit-setting stage (September), the seed colour was inspected in well-developed plants by sampling 20–30 young fruits from different positions of the inflorescence every 4 days. For comparison, from August to September, fruits from the natural habitat of *S. aralocaspica* in a wild field were also sampled every week and the same analysis was carried out.

### Analysis of seed heteromorphic ratio in *S. aralocaspica*

Seeds from different sizes of plants were sampled in the natural habitat in October from 2008 to 2012. The average plant sizes were defined according to the plant height × length × width as: large (25–30 × 55–65 × 40–50 cm), medium (20–25 × 40–50 × 25–35 cm) and small (15–20 × 20–30 × 15–20 cm). There were some changes among different calendar years depending on the weather conditions. All mature fruits from 10 individual plants were harvested and mixed to determine the seed heteromorphic ratio; and the fruits from 10 individual plants at different positions on the inflorescence were separately harvested to determine the seed heteromorphic ratio. All harvested fruits were air-dried indoors before examination.

During fruit maturation (in September, the colour of most fruits changed from green-yellowish to yellow, and fruits at the top of the branch were becoming dry), three sampling plots measuring 2 × 2 m^2^ each and 5–6 plants per plot were randomly selected from the same habitat, the above-ground part of the individual plant was carefully harvested and seeds were air-dried indoors and cleaned. Four replicates with 1000 seeds in each were randomly sampled from the mixed intact seeds of the same habitat to calculate the seed heteromorphic ratio.

### Quantitative PCR analysis of the expression pattern of seed development-related genes

**Figure 1.**
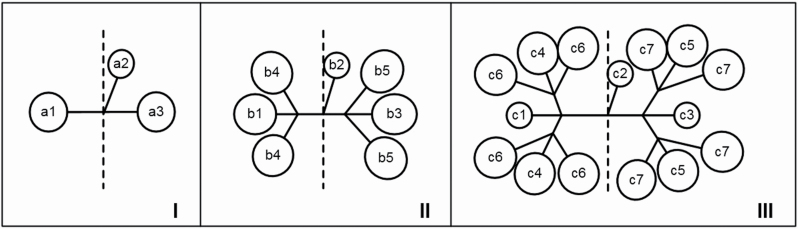
Schematic diagram of seed-setting patterns (SSP) in *S. aralocaspica*. I, II, III: representative SSP of the upper, middle, lower part of the inflorescence. a, b, c: seed location corresponding to I, II, III; 1, 2, 3, 4, 5, 6, 7: seeds in different positions at the same location on the inflorescence.

Total RNA was isolated from 0.10 to 0.15 g fresh fruits of *S. aralocaspica* according to the manufacturer’s instructions using the General Plant Total RNA Extraction Kit (BioTeke, Beijing, China). The fruits were sampled at 1, 9, 18 and 27 days after pollination (DAP) from different positions or locations on the inflorescence (a1 + a3, a2 + b2, b1 + b3, b4 + b5), as shown in Fig. 1. Each reverse transcription reaction was performed at 42 °C for 1 h with 1.0 μg of total RNA in a volume of 20 μL using the reverse transcriptase M-MLV (TaKaRa, Dalian, China) according to the manufacturer’s instructions.

Quantitative real-time PCR (qPCR) was carried out using the GeneAmp 5700 Sequence Detection Real-time PCR System (ABI, USA) and SYBR Green (Invitrogen, USA). *SaANS* (*Anthicyanidin synthase*), *SaBAN* (*Banyuls*), *SaTT12* (*Transparent testa* 12) and *SaABI3* (*Abscisic acid-insensitive* 3) genes were amplified with the specific primers described in **Supporting Information—Table S2**. The relative amplification of the *β-actin* gene of *S. aralocaspica* was used for normalization (Cao *et al.* 2016). The relative expression level of the target genes was quantified according to the equation: *R* = 2^−∆∆*C*^_T_ (Shi and Chiang 2005). Three biological replicates with two technical replicates of each sample were included. The final value of relative quantification was described as fold change of gene expression in the test sample compared to the control.

### Seed germination experiments

Mature intact seeds collected from 2008 to 2012 were used in seed germination experiments (most experiments used seeds from 2008 unless noted otherwise). Three replicates with 30 seeds in each replicate were included in each treatment. For germination, different types of seeds were sown on two layers of double-distilled water-saturated filter paper in a 9-cm Petri dish, in a constant temperature and humidity incubator (LRH-400-GII light incubator; Medical equipment factory in Guangdong, China) to germinate (16 h light/8 h dark; 25 °C; RH 30–40 %; light intensity is ~116 μmol m^−2^ s^−1^). A seed was considered to be germinated when the radicle protruded from the seed coat for more than half the length of the seed. Germination was recorded every 24 h for 2 weeks. The final germination percentage was calculated on the 15th day. To determine the effect of different seed positions on germination, seeds from 2011 were collected separately from different positions on the inflorescence of individual plants according to the schematic diagram of Fig. 1, and after air-drying and cleaning, different types of seeds from different positions were tested in germination experiments separately. To determine the effect of black seed coat on germination, the seed coat of large black seeds was carefully peeled off the seed using a dissection needle; the black small seeds were squeezed before carefully removing the seed coat from the seed hilum site using a dissection needle, without injuring the radicle. The seed embryos were then sown to allow germination. To determine the effect of darkness on seed germination, the Petri dishes with sown seeds were wrapped with foil paper and placed in normal conditions as above mentioned. The foil was uncovered 14 days later to calculate the germination percentage. To determine the effect of long-term storage on germination, brown and black seeds collected in 2010 were dry stored in brown paper bags without disturbance and placed in an incubator at room temperature (20–28 °C, 15–25 % RH) or at 4 °C in a refrigerator. The germination experiment was carried out at the beginning of each month from July 2011 to October 2012. For determination of seed viability, after germination for 15 days under different treatments (300 mmol·L^−1^ NaCl, 20 % PEG and 4 °C), the seed coat of ungerminated large and small black seeds was removed and the embryos were treated with a 1 % solution of 2,3,5-triphenyltetrazolium chloride (TTC) (Baskin and Baskin 1998), then checked for seed viability under the stereomicroscope. Seeds with the greatest viability were stained dark red, viable seeds stained red and inactive seeds showed no staining. Three replicates with 15 seeds each from the ungerminated seeds were stained and observed.

### Statistical analysis

All data were expressed as mean ± SE. One-way ANOVA was used to test the significance of different treatments, and Tukey’s HSD test was performed for multiple comparisons to determine significant differences between the samples at the 0.05, 0.01 and 0.001 significance level. When the homogeneity of variance assumption was not met, tests for differences were performed with Welch’s ANOVA and Games Howell *post hoc* test. All proportions (e.g. germination and hetero-seed percentage) were arcsine transformed before statistical analysis to ensure homogeneity of variance (non-transformed data appear in all figures). Chi-square test for independence was used in seed viability analysis. Correlation analysis was performed to identify the associations between meteorological factors and seed heteromorphic ratio or seed number of individual plants. All data were plotted using the software of GraphPad Prism Version 5.02 for Windows (GraphPad Software, San Diego, CA, USA) and analysed using SPSS Version 26.0 for Windows (SPSS Inc., Chicago, IL, USA).

## Results

### Morphology and formation of heteromorphic seeds in *S. aralocaspica*

**Figure 2.**
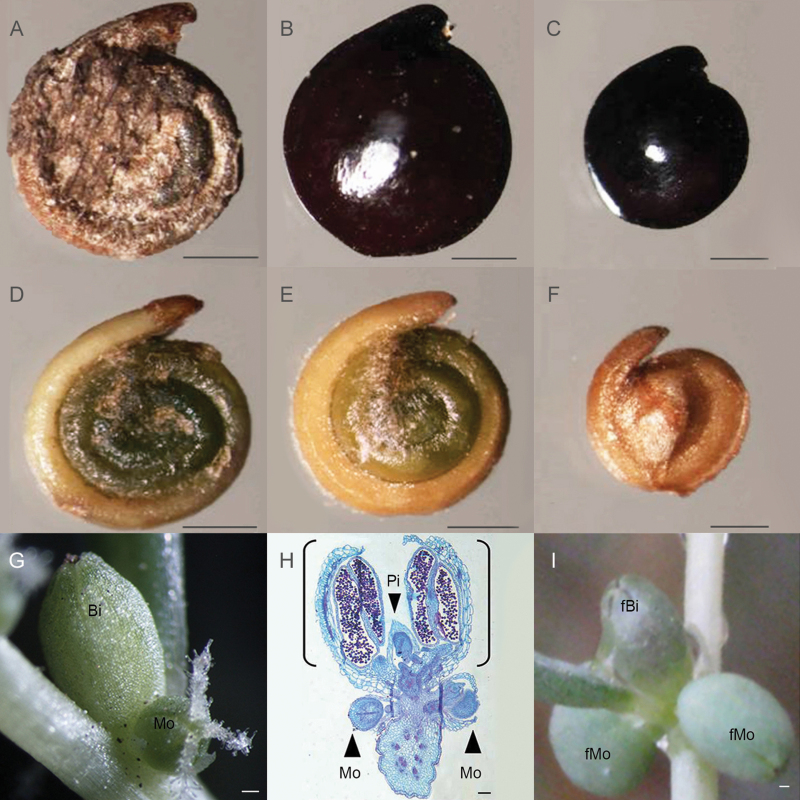
Morphology and origin of different types of seeds in *S. aralocaspica*. A, D: brown seed; B, E: large black seed; C, F: small black seed; G: two types of flower in leaf axil; H: paraffin section showing seeds developed from two types of flower, parts in the bracket represent bisexual flower; I: fruits developed from two types of flower. Bi: bisexual flower; Mo: monosexual flower; Pi: pistil; fBi: fruit from bisexual flower; fMo: fruit from monosexual flower. Scale bar in A–F is 1 mm; in G, I scale bar is 500 μm; in H scale bar is 50 μm.

A large number of observations and measurements were made of the heteromorphic seeds from 2008 to 2012, revealing two shapes and colours: flat round, brown seeds and oblate black seeds, the latter having two different sizes, large and small (Fig. 2A–C). Removal of the seed coat revealed the diverse appearance of the embryo. All of the brown seeds were dark green, and most of the large black seeds were green, while most of the small black seeds were yellow (Fig. 2D–F).

Measurement of the size and the thousand-seed weight (TSW) showed that the largest seeds were the brown seeds (2.604 ± 0.277 mm in diameter; 2.260 ± 0.123 g in TSW), and the smallest were the small black seeds (1.880 ± 0.022 mm in diameter; 1.397 ± 0.025 g in TSW). There were significant differences between the brown and the large black seeds (*P* < 0.05), and also between the large and small black seeds (*P* < 0.05) (Table 1).

**Table 1.** The morphological characteristics and thousand-seed weight of the brown, large black and small black seeds. Different lowercase letters in each column indicate significant differences (*P* < 0.05) among different seed types.

Seed type	Color	Shape	Diameter (mm, mean ± SE)	Thickness (mm, mean ± SE)	Thousand-seed weight (g, mean ± SE)
Brown	Brown	Flat round	2.604 ± 0.277^a^	0.670 ± 0.020^c^	2.260 ± 0.123^a^
Large black	Black	Oblateness	2.257 ± 0.057^b^	1.280 ± 0.033^a^	2.183 ± 0.024^b^
Small black	Black	Oblateness	1.880 ± 0.022^c^	1.120 ± 0.025^b^	1.397 ± 0.025^c^

Observation under the stereomicroscope and of paraffin sections revealed that the size difference between the large and small black seeds was due to the type of flower from which they originated (Fig. 2). Our results revealed that, in this monoecious species, there were not only monosexual (unisexual) flowers but also bisexual flowers (considered as the ‘male flower’ by the Commissione Redactorum Florae Xinjiangensis 1994) in which an incomplete degenerated pistil was present and might be able to develop into a small black seed (Fig. 2G–I; Bi, Pi, fBi). This was usually located in the middle of two unisexual female flowers (Fig. 2G–I; Mo, fMo). This middle fruit (seed) was much smaller and had a very short carpopodium, which caused the fruit to easily abscise from the leaf axil.

### Effects of genetic aspects on formation of seed heteromorphism in *S. aralocaspica*

#### Seed-setting patterns.

**Figure 3.**
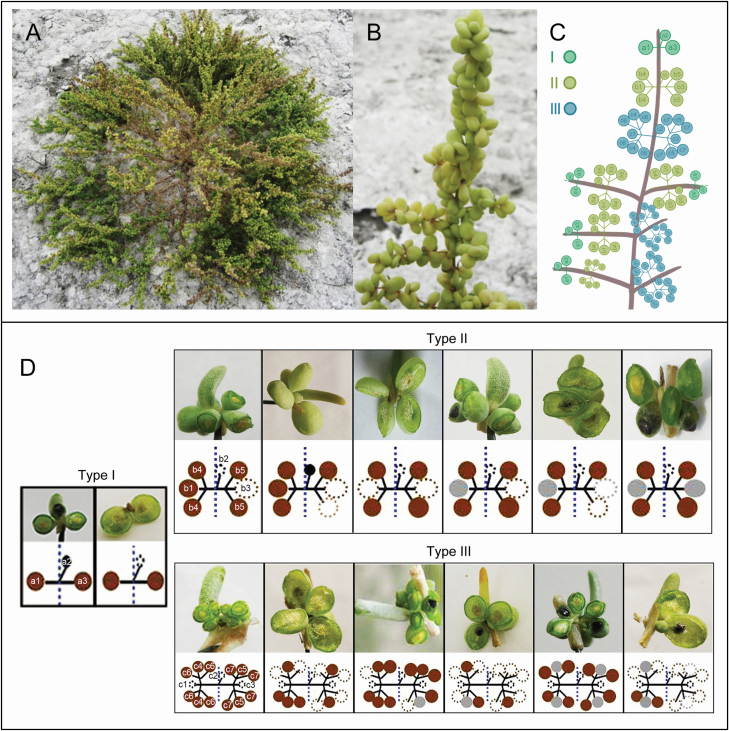
Morphology and schematic diagram of different seed-setting patterns (SSP) in *S. aralocaspica*. A: a fully developed plant in fruiting stage in natural habitat; B: fruit branch; C: illustration of three types of SPP on the fruit branch; D: the SSP on different dichasium inflorescences, the upper panel in each type shows samples collected from the natural habitat; the lower panel shows the schematic diagram of SSP corresponding to Fig. 1. Type I, II, III: corresponding to I, II, III in Fig. 1. Blue dashed line represents inflorescence axis; black solid line represents fruit stalk; black solid circle represents small black seed; brown solid circle represents brown seed; gray solid circle represents large black seed; dotted open circle represents a probable setting seed or shed seed.

 From 2008 to 2012, a large number of observations of flowering behaviour and seed-setting pattern of the inflorescence were made (Fig. 3A and B). This revealed that the *S. aralocaspica* flower forms a dichasium (Fig. 3C and D; Type I, II, III). Generally, the bisexual flower is located in the middle and female flowers are located on both sides of the leaf axil. The former blossomed first followed by the latter, the flowering order of the inflorescence was from the lower part to the upper part, each flower was open for 3–5 days and the flowering time of the whole inflorescence lasted from the end of July to late August.

Based on many years of field investigations and inspection of the paraffin sections in the lab, we found that the ovary in the bisexual flower of *S. aralocaspica* is not completely degenerated (Fig. 2), although it has traditionally been considered as the male flower by the Commissione Redactorum Florae Xinjiangensis (1994). It could develop into a normal small black seed that abscises easily upon maturation. However, only a few positions, e.g. a2, b2, c1, c2, c3 in Fig. 3D (Type I, II, III) could develop into a small black seed from a bisexual flower. The female flowers in other positions on the inflorescence would develop into large black seeds or brown seeds. The seed-setting pattern can be roughly divided into three categories corresponding to the different parts on the inflorescence, according to observations of fruit maturation and statistical analysis of the seed colour. In the upper part, three seeds would form in the leaf axil, a small black seed might develop from a bisexual flower in the middle position (e.g. a2) and one brown seed was usually formed on each side, where a large black seed might also be produced in some cases [Fig. 3D (Type I)]; in the middle part of the inflorescence, another three seeds would develop on each side of the middle small black seed (e.g. b2) [Fig. 3D (Type II)]; in the lower part of the inflorescence, the seed-setting pattern was more complex as shown in Fig. 3D (Type III). There were more seeds on each side of the middle small black seed, in which c1, c2, c3 were most likely the small black seeds and the others would be brown or large black seeds; the other middle position (e.g. c4, c5) usually but not always bore a large black seed. When the main inflorescence was fully developed with secondary fruit branches measuring 3–5 cm or even longer, the individual plant could yield thousands of seeds; in this case, the number of brown seeds would be significantly higher than that of black seeds; however, if the main inflorescence was poorly developed without or with fewer secondary fruit branches measuring 1–2 cm long, the total number of seeds was significantly reduced, and the black seed ratio increased significantly, because the seed in the middle position could be the first to form and develop.

#### Developmental cytology of heteromorphic seeds.

**Figure 4.**
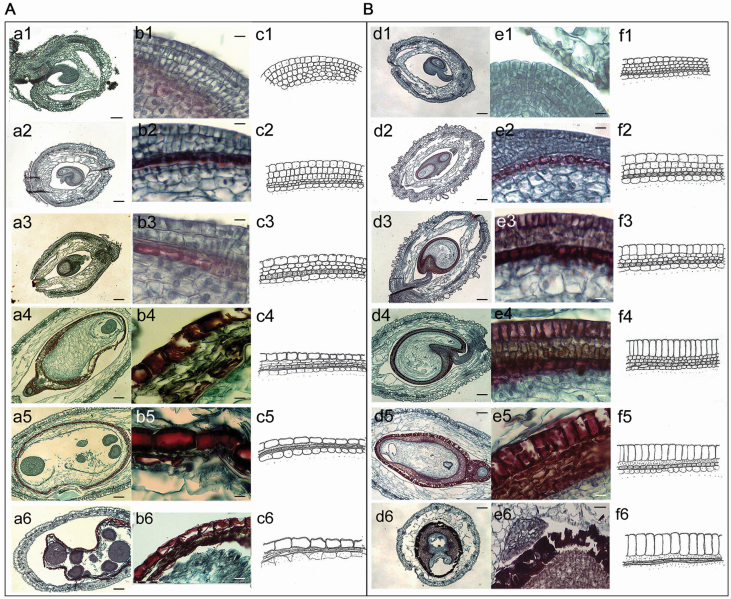
Cytological process of seed coat development of brown and black seeds in *S. aralocaspica.* A: brown seed; B: black seed. 1–6: different developmental stages. a, d: embryo development; b, e: corresponding seed coat development; c, f: schematic diagram of seed coat development. Scale bar in a1, e6 is 100 μm; in a2–a6, d1–d6 scale bar is 500 μm; in b1–b5, e1–e5 scale bar is 50 μm; in b6 scale bar is 250 μm.

 A large number of paraffin sections were prepared to observe seed development under a light microscope. Following the completion of fertilization, the inner and outer integuments of the mature ovule began to differentiate into the inner and outer seed coats. The cytological structure showed a significant difference between brown and black seeds (Fig. 4). The whole developmental process of heteromorphic seeds could be divided into four stages. In the first stage, the fertilized embryo sac initiated development in the ovary but the cell shape and size of the seed coat did not differ significantly between brown and black seeds, in which a large nucleus and thick cytosol were present [Fig. 4A (a1–c1); Fig. 4B (d1–f1)]. In the second stage, the epidermal cells of the outer integument of both types of seeds began to expand and differentiate into the early seed coat; layers of inner integument were gradually reduced. Cells in the outermost integument layer began to extend in different directions [Fig. 4A (a2–c2, a3–c3); Fig. 4B (d2–f2, d3–f3)]. In the third stage, the epidermal cells of the brown and black seeds showed a significant difference, expanding in the tangential direction and being loosely arranged in the brown seeds [Fig. 4A (a4–c4, a5–c5)] but expanding in the radial direction and being tightly arranged in the black seeds [Fig. 4B (d4–f4, d5–f5)]. The cell wall was significantly thickened. In the fourth stage, when the embryo was becoming mature, the testa cell layer of the brown seeds became thin and loosely arranged [Fig. 4A (a6–c6)], whereas that of the black seeds was apparently lignified and hardened [Fig. 4B (d6–f6)]. Our results suggest that the differentiation of the epidermal layer of the seed coat takes place in the early stages of embryo development, which showed significant differences between the two types of seed.

#### Expression patterns of seed development-related genes.

**Figure 5.**
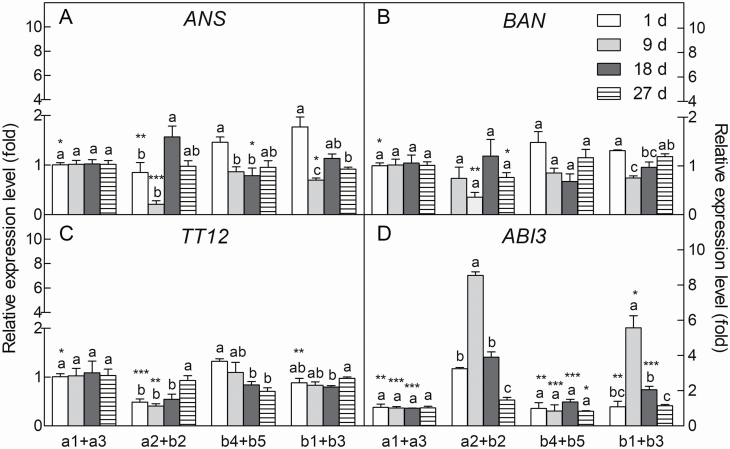
Quantitative PCR analysis of expression patterns of *SaANS*, *SaBAN*, *SaTT12* and *SaABI3* genes in seeds from different positions and different development times. A, B, C, D: different genes; a1 + a3, a2 + b2, b4 + b5, b1 + b3: different seed positions corresponding to Fig. 1; 1 day, 9 days, 18 days, 27 days: day(s) after pollination (DAP). *SaANS*: anthocyanin synthase gene; *SaBAN*: anthocyanidin reductase gene; *SaTT12*: transparent testa 12 gene; *SaABI3*: abscisic acid-insensitive 3 gene. Different lowercase letters of the same position indicate significant differences (*P* < 0.05) among different DAP; *, **, ***: indicate significance existing in the same DAP among different positions at 0.05, 0.01, 0.001 level. Values are means ± SE of six replicates.

 To gain insight into the reason for the difference between the brown and black seeds, the expression patterns of four genes related to seed (or seed coat) development were analysed by qPCR. The transcript accumulation of *SaANS*, *SaBAN*, *SaTT12* and *SaABI3* genes was analysed in seeds located at four different positions on the inflorescence (which corresponded to positions shown in Fig. 1) during seed development. Based on our investigation, seeds in position a2 or b2 were most likely to develop into small black seeds, those in position a1, a3, b4 or b5 were usually brown seeds and those in b1 or b3 were likely to develop into large black seeds. The expression pattern of *SaBAN* gene did not differ significantly between positions a2 + b2 and b4 + b5, but the expression of *SaANS* and *SaTT12* genes was apparently different, i.e. the transcripts accumulated in positions a2 + b2 but declined significantly in positions b4 + b5 during seed development. The expression of *SaANS* for positions b4 + b5 and b1 + b3 was similar in that they both had higher accumulation early in development (1 day) and then levels dropped sharply (from 9 days onwards) (Fig. 5A–C). *SaABI3* gene (related to seed development and dormancy) was significantly upregulated in seeds at positions a2 + b2 and b1 + b3 (usually yielding black seeds) (Fig. 5D) in comparison with positions a1 + a3 or b4 + b5 (usually yielding brown seeds); the expression level was 8-fold higher at positions a2 + b2 and ~6-fold higher at positions b1 + b3 than the other two positions on the 9th day after pollination.

### Effects of environmental factors on seed heteromorphism in *S. aralocaspica*

#### Regular pattern and fluctuation of natural habitat conditions among different calendar years.

**Figure 6.**
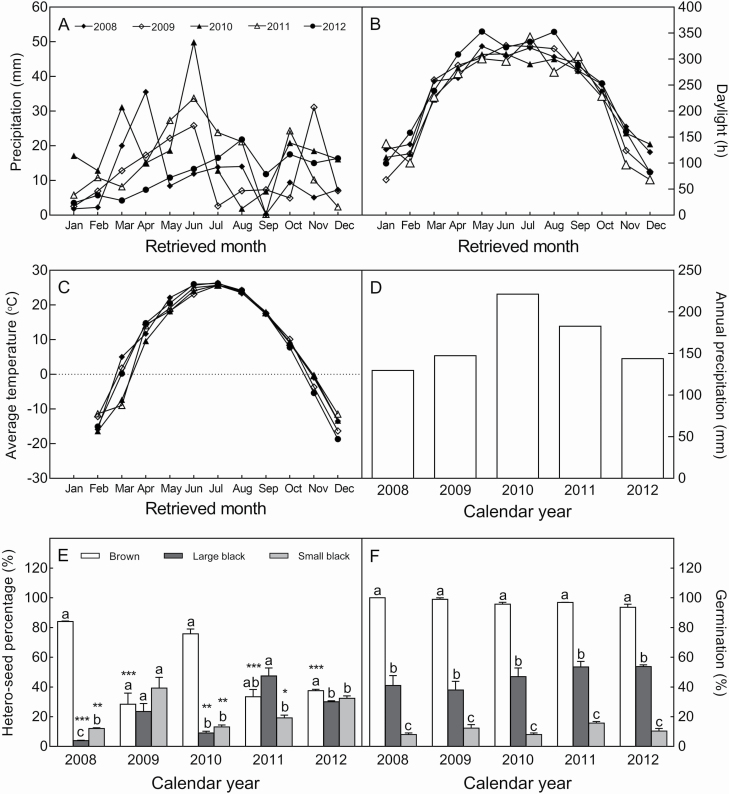
Fluctuation of environmental conditions and variations in seed heteromorphism in *S. aralocaspica* during over 5 years (2008–12). A: precipitation per month (PM); B: hours of daylight per month (HDL); C: average temperature per month (ATM); D: annual precipitation (AP); E: heteromorphic seed ratio; F: seed germination of heteromorphic seeds. Different lowercase letters in the same calendar year indicate significant differences (*P* < 0.05) among different seed types; *, **, ***: indicate significance existing in the same seed type among different calendar years at 0.05, 0.01, 0.001 level. Values are means ± SE of three replicates with 500 seeds in each in E; three replicates with 30 seeds in each in F.

A 5-year (2008–12) investigation of the natural field conditions showed that the precipitation per month (PM), annual precipitation (AP), average temperature per month (ATM) and hours of daylight per month (HDL) showed some fluctuation (Fig. 6), in which the variation in PM and AP was significant and irregular (Fig. 6A and D), whereas that of HDL and ATM was relatively mild and regular among different years (Fig. 6B and C). The PM from March to May was higher in 2008 and 2010; the ATM was lower in 2010 and 2011; the AP was higher in 2010 and 2011.

#### Variation of heteromorphic ratio.

 The results showed that the heteromorphic seed ratio differed significantly among different calendar years (Fig. 6E): 2008 and 2010 showed a similar pattern, i.e. the brown seed accounted for ~80 % and the two black seed types only for ~20 % of the total number; however, in 2009, 2011 and 2012, the large and small black seeds accounted for a high proportion (60–70 %) of the total seed number. No significant difference was observed regarding seed germination of the same seed type among different years (Fig. 6F); however, seed germination varied widely among the three seed types. Based on the analysis of HDL, ATM and PM, only PM showed a significant fluctuation over the whole year. The large proportions of brown seed in 2008 and 2010 (84.03 % and 75.75 %, respectively) coincided with the large amount of precipitation from March to May (the cumulative PM in 2008 and 2010 was 63.9 mm and 64.7 mm, respectively), indicating that the variation in seed morph ratio may depend on the precipitation in Spring. The relatively higher PM and AP were significantly and positively correlated with the total number of seeds per plant (*r* = 0.718 and 0.717, *P* < 0.05), and with producing more brown seeds (*r* = 0.800, *P* < 0.01), but were significantly and negatively correlated with the small black seed ratio (*r* = −0.855, *P* < 0.01), which was also significantly and positively correlated with the average ATM (*r* = 0.726, *P* < 0.01) (Table 2).

**Table 2.** The correlation coefficients between seed heteromorphic ratio and meteorological data in 2008–12. AP: annual precipitation; ATM: average temperature per month; HDL: hours of daylight per month; PM: precipitation per month; TSP: total seeds per plant. *, **: indicate significance at 0.05 and 0.01 level.

Seed type	Average PM (from March to May)	AP	Average HDL	Average ATM
Brown	0.473	0.800**	−0.033	−0.508
Large black	−0.424	−0.428	−0.153	−0.090
Small black	−0.505	−0.855**	0.398	0.726**
TSP	0.718*	0.717*	−0.541	−0.674*

**Figure 7.**
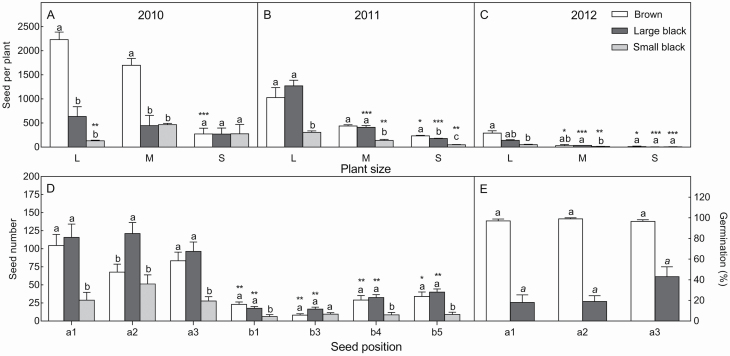
Variations in heteromorphic seed number in individual plants and at different positions on the inflorescence and corresponding germination behaviour. A–C: heteromorphic seed number of large-, middle- and small-sized plants in 2010, 2011, 2012 calendar years; D: heteromorphic seed number at different positions on inflorescence in 2011; E: germination of seeds in different positions. a1–a3, b1–b5: represent different positions on inflorescence, corresponding to Fig. 1. In A–D, different lowercase letters for the same plant size (or seed position) indicate significant differences (*P* < 0.05) among different seed types; *, **, ***: indicate significance existing in the same seed type among different plant sizes (or positions) at 0.05, 0.01, 0.001 level. In E, different normal or italic lowercase letters indicate significant differences existing within the same seed type (*P* < 0.05) among different positions. In A–D: values are means ± SE of 10 plants; in E: values are means ± SE of three replicates with 30 seeds in each.

To understand the details of the variation in seed morph ratio, we investigated the heteromorphic seed number in individual plants and at different positions on the inflorescence over 3 years (Fig. 7). The results showed that the total seed number per plant was the highest in 2010 and the lowest in 2012 (Fig. 7A–C), corresponding to the amounts of AP (Fig. 6D); moreover, precipitation significantly affected the heteromorphic seed ratio, with brown seeds being more numerous in 2010 (Fig. 7A) compared to 2011 and 2012 (Fig. 7B and C). Plant size also influenced the heteromorphic seed ratio (Fig. 7A). According to the seed-setting pattern (Fig. 3), when a plant was well developed in the vegetative growth and reproductive stage, the total number of seeds per plant was higher, and the number of brown seeds was significantly higher than that of black seeds. The seed number at different positions on the inflorescence in 2011 showed that all positions had more black seeds (large and small) than brown seeds (Fig. 7D). However, the same seed type from different positions showed no significant differences in germination behaviour (Fig. 7E).

### Effects of seed coat, light and long storage on seed germination in *S. aralocaspica*

**Figure 8.**
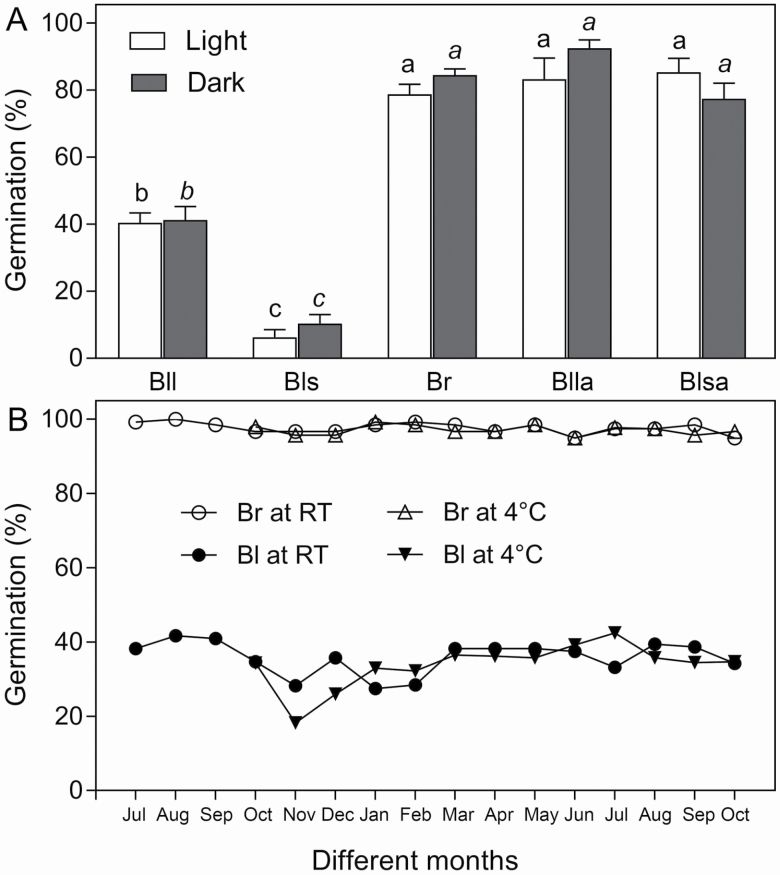
Effects of seed coat, light and long-term dry storage on seed germination. A: germination behaviour of seeds with or without seed coat under different light treatments; B: monthly germination of seeds in long-term storage under different temperatures. Germination was carried out at the beginning of each month from July 2011 to October 2012 using seeds collected in October 2010. RT: room temperature; Br: brown seed; Bll: large black seed; Blla: seed coat removed Bll; Bls: small black seed; Blsa: seed coat removed Bls. Different normal or italic lowercase letters indicate significant difference (*P* < 0.05) existing within the same light or dark treatment among different seed types. Values are means ± SE of four replicates with 30 seeds in each.

After removal of the seed coat, the large and small black seeds showed a similar germination percentage under light (>80 %) (Fig. 8A), while in the dark the germination percentage of black small seeds was slightly lower than that of the brown (around 85 %) or the large black (around 90 %) seeds.

During 2 years (from October 2010 to October 2012) of dry storage at room temperature or 4 °C, monthly germination tests revealed that at both temperatures, the germination percentage of brown seeds was higher (near 100 %) than that of black seeds (around 40 %) (with some fluctuation) (Fig. 8B). No significant decline in the germination percentage was observed over the 2 years, and the storage temperature (room temperature or 4 °C) had no significant effect on seed germination within the tested period. After being tested with TTC staining, 90 % of the ungerminated black seeds were confirmed as viable at the end of the germination experiment **[see**  **Supporting Information—Fig. S1****]**.

## Discussion

More than 200 species have been identified to have seed heteromorphism under unpredictable environments (Imbert 2002). *Suaeda aralocaspica* is an annual halophyte with heterospermy growing in inland cold deserts, and can produce different seed types with diverse characteristics of dormancy (Wang *et al.* 2008). However, the major factors influencing the formation and variation of seed heteromorphism in *S. aralocaspica* have not been well understood so far. In the present study, we demonstrated that both genetic (internal) and maternal environmental (external) factors have significant effects on seed heteromorphism and its variations. Our investigations involved redefining the seed heteromorphs, seed ontogeny and variations in seed heteromorphism, in which we found a third type of seed in *S. aralocaspica* in addition to the two previously reported types (Commissione Redactorum Florae Xinjiangensis 1994), and revealed the expression profiles of four seed development- and dormancy-related genes. These data suggest an accommodation mode combined with the bet-hedging and plasticity strategy of heterospermy in *S. aralocaspica*, which has important ecological significance for the adaptation mechanism of such desert plant species in heterogeneous habitats.

*Suaeda aralocaspica* has been documented as showing seed heteromorphism (Liu *et al.* 2009; Wang *et al.* 2012a, b, 2016, 2017a, b; Song *et al.* 2012; Cao *et al.* 2015, 2016), with reports that the two types of seed (brown and black) present significant differences in morphology, germination properties (Wang *et al.* 2008), tolerance to abiotic stresses (He *et al.* 2013; Song *et al.* 2014), etc. In the present study, however, we discovered a third type of seed of *S. aralocaspica*, which has not been documented previously. Besides the brown seed, two distinct sizes of black seeds were further distinguished among seeds developing from bisexual and pistillate flowers, respectively. The bisexual flower was considered as monosexual staminate in previous work (Commissione Redactorum Florae Xinjiangensis 1994), but we found that a non-completely degenerated ovary would develop into a small black seed, while the pistillate flower would produce a large black or brown seed. These three distinct seed types (brown, large black and small black) differed in seed size, mass and dormancy properties. The non-dormant brown seeds of *S. aralocaspica* have potential advantages in favourable conditions, whereas the black seeds can compensate for the loss of individuals under extreme conditions (Wang *et al.* 2008). Regarding dormancy, a large proportion of the small black seeds of *S. aralocaspica* may enter the soil seed bank for long-term needs in unpredictable circumstances (Ungar 1987). Moreover, the three types of seed presented different dispersal abilities on seed maturation, as also observed in *Ceratocarpus arenarius* and *Salsola ferganica* (Lu *et al.* 2013; Ma *et al.* 2018). The utricles (Commissione Redactorum Florae Xinjiangensis 1994) of brown and large black seeds are larger and have more empty space in the dry, vesicular pericarp, which facilitates seed dispersal by wind over a wide area (Wang *et al.* 2012b); the small black seed with the pericarp tightly enclosing the seed together with the short carpopodium makes the smaller utricle more easy to shed and disperse nearby the mother plant. Taken together, we speculate that the three types of heteromorphic seeds of *S. aralocaspica* allow germination under diverse conditions in response to heterogeneous environments. Such a multilevel combination of strategies should be evolutionarily advantageous for this species surviving and thriving in the extreme habitats.

Different positions of fruits or seeds on the inflorescence can apparently affect seed colour, size, mass and dormancy characteristics (Venable and Levin 1985; Gutterman 2000; Imbert 2002; Talavera *et al.* 2010). These phenomena are considered as ‘position-dependent effects’ (Cheplick and Sung 1998; Moravcová *et al.* 2005). In the present study, we found that three primary and 15 secondary types of seed-setting patterns existed on the fully developed inflorescence of *S. aralocaspica*, in which seed morphs showed apparent position effects, i.e. the middle position tended to produce more large black seeds (accounting for 63.82 % of seeds in positions b1 + b3 in 2011), while the side position tended to produce brown seeds (however, in 2011 brown seeds only accounted for 41.36 % of seeds in positions a1 + a3 + b4 + b5, because due to unfavourable weather conditions, the total number of seeds was much lower and black seeds accounted for a higher proportion), and the central middle position tended to produce small black seeds (accounting for 21.36 % in position a2, much higher than the average ratio of 12.65 % in other positions). It could be speculated that, when the natural conditions are favourable, the inflorescence of an individual plant may develop fully and a large number of seeds would be expected, with brown seeds (at the side position) accounting for the majority of the total seeds (e.g. 2010 in the present study); when the inflorescence is poorly developed, the two types of black seeds (at the middle or central middle position) account for a higher proportion of seeds and the total seed number would be lower (e.g. 2011 in the present study). Such a seed ontogeny could be a strategy of *S. aralocaspica* to accommodate unpredictable heterogeneous environments (Baskin and Baskin 1998; Moravcová *et al.* 2005; Wang *et al.* 2010). Various studies have focused on the germination characteristics of seeds that develop from different positions on the inflorescence, including species in Asteraceae (Foroughi *et al.* 2014), Poaceae (Dyer 2004; Wang *et al.* 2010), Apiaceae (Moravcová *et al.* 2005) and Gramineae (Volis 2016), and these suggest that variations in germination or dormancy may occur according to position (Datta *et al.* 1970), even within a single capsule or pod (Gutterman 2000). In the present study, however, the germination of a particular seed type (brown or black) was independent of position. Taken together, our work suggests that variations in the seed morphs of *S. aralocaspica* are associated with the positions of seeds on the inflorescence, while the difference in dormancy seems to be closely related to the heteromorphic seed types rather than the seed’s position on the inflorescence.

The formation of distinct structures in different types of seeds and the expression of relevant genes in development are what make the maternal position effect possible (Gutterman 2000; Lenser *et al.* 2016). Seed develops from the ovule, and the differentiation (e.g. thickness and contour changes, pigment deposition) of integument layers at the proper time is critical for the commitment of heteromorphic seeds (Li *et al.* 2016). In the present study, our cytological results indicate that the differentiation of brown and black seeds began in the early stages of embryo development, in which the outermost integument cell layer became radially extended and tightly arranged with thickened cell walls in the black seed, but tangentially extended and loosely arranged without cell wall thickening in the brown seed. However, heteromorphic seeds differ not only in seed coat structures, but also in seed coat colour and dormancy characteristics (Yao *et al.* 2010; Cao *et al.* 2012; Baskin and Baskin 2014). The distinct colours of seed coat may involve different levels of pigment deposition (Doughty *et al.* 2014), e.g. the black seed of *S. salsa* might contain different levels of pigments than the brown seed (Li *et al.* 2005). Several genes of the maternal plant may influence seed coat coloration, e.g. *ANS* (anthocyanidin synthase) (Zhao *et al.* 2019), *BAN* (procyanidin-synthesizing enzyme) (MacGregor *et al.* 2015) and *TT12* (transparent testa) (Chai *et al.* 2009). In the present study, three seed colour-related genes, *SaANS, SaBAN* and *SaTT12*, presented different expression patterns among seeds from different positions on the inflorescence. These genes were upregulated in the developing seeds at positions that would yield black seeds, and downregulated in seeds at positions yielding brown seeds (especially expression of *SaTT12*), which suggests that pigment deposition tends to be more active in black seed development. More interestingly in the present study, *SaABI3*—a seed dormancy-related gene (Baumbusch *et al.* 2004)—showed a significantly higher expression level in positions yielding black seeds compared to those of brown seeds. Furthermore, seeds at positions yielding small black seeds accumulated much higher levels of *SaABI3* transcripts than seeds at positions yielding large black seeds, which was consistent with the much lower germination percentage of the small black seeds. It is considered that, besides the thicker and harder seed coat of black seed, the relevant gene expression might be another essential factor resulting in delayed germination (Li *et al.* 2005; Li and Li 2015; Yao *et al.* 2010). Our results suggest that the active expression of *SaABI3* is likely to be important in regulation of the dormancy condition among different types of seeds in *S. aralocaspica*. Taken together, obvious differences existed among brown, large black and small black seeds of *S. aralocaspica* during development, i.e. seed coat structural change and coloration progression, and dormancy-related gene regulation; all these aspects would contribute to the genetic (internal) factors by which the formation of seed heteromorphs in *S. aralocaspica* may come into being.

Adaptive phenotypic plasticity is a phenomenon whereby one genotype produces different phenotypes depending on the environmental situation (Robinson and Wilson 1996), which allows plants to have higher fitness. The variations in seed heteromorphism among different calendar years may be an effect of phenotypic plasticity on the seed morph ratios of an individual plant (Venable *et al.* 1987; Sadeh *et al.* 2009; Lu *et al.* 2012). In the present study, the seed heteromorphism of *S. aralocaspica* varied significantly over 2008–12, which was reflected in variations of total seed number per plant and the morph ratios among different seed types, and both were closely associated with the fluctuations in environmental conditions (Table 2). Based on the developmental rhythms, in the natural habitat, *S. aralocaspica* seeds germinate at the end of March and seedlings are established during April and/or May, then flower and fruit from early August to September. Analysis of the correlation between seed heteromorphism variations and the meteorological data (2008–12) revealed that higher AP combined with lower ATM was correlated with a higher proportion of brown seeds and fewer small black seeds. The PM from March to May also appeared to have a considerable effect on seed morph ratio (Table 2). A similar phenomenon has been observed in *Aethionema arabicum*, in which natural temperature and moisture gradients significantly affect the plasticity of life phases and the fruit morph ratio of this species (Bhattacharya *et al.* 2019). In addition, lower AP resulted in the establishment of smaller sized plants and fewer total seeds per plant in *S. aralocaspica*, as also reported in *S. corniculata* subsp. *mongolia* (Yang *et al.* 2015a). Our data, in accordance with several other studies (Imbert and Ronce 2001; Sadeh *et al.* 2009; Lu *et al.* 2012), suggest that different maternal environmental conditions significantly contribute to the variations in seed heteromorphism of *S. aralocaspica*. Such combined effects of phenotypic plasticity and seed heteromorphism form an advanced adaptation strategy of *S. aralocaspica* to deal with unpredictable environmental changes. However, the physiological mechanism of this phenomenon needs to be further explored.

Seeds of desert plants can persist in the soil seed bank for a long time without significantly altering seed viability (Gremer *et al.* 2016; Jaganathan 2016), which can help survival until the next favourable conditions for germination (Jayasuriya *et al.* 2012; El-Keblawy and Bhatt 2015). In the present study, during 2011–12 and the following 5 years (data not shown), the germination percentage of the heteromorphic seeds of *S. aralocaspica* remained relatively high (the brown) and stable, while ungerminated black seeds remained viable **[see**  **Supporting Information—Fig. S1****]**. Furthermore, during long-term storage the germination did not seem to be significantly affected by varying environmental conditions, e.g. the storage temperature (4 °C or 25 °C), with or without exposure to light, etc., suggesting that such properties of seeds may be essential for supporting the individual ontogeny and population succession in desert habitats (Gutterman 1993).

## Conclusions

In the present study, we show that the heterospermy of *S. aralocaspica* consists of three types of seeds (brown, large black and small black), instead of the originally reported brown and black seeds, with significant differences in dormancy properties and ecological significance in individual ontogeny and population succession. We further demonstrate that the seed heteromorphism of *S. aralocaspica* and its variations among different calendar years can be attributed to a combination of genetic (internal) and environmental (external) factors. Seed coat structure and coloration and seed dormancy are predominantly influenced by genetic (internal) aspects during seed development. Meanwhile, the maternal environmental (external) conditions could have a significant influence on variations in plant size, seed number, heterospermic ratios, etc. Our data suggest that a comprehensive strategy in the seed stage may ensure *S. aralocaspica* survival in unpredictable circumstances, which is also important for understanding the adaptation mechanism of this and other desert plant species.

## Supporting Information

The following additional information is available in the online version of this article—

**Figure S1.** Seed viability and germination characteristics in *Suaeda aralocaspica*.

**Table S1.** The components and the physical and chemical properties of the soil in the natural habitat of *Suaeda aralocaspica.*

**Table S2.** Quantitative PCR (qPCR) primers of *SaANS*, *SaBAN*, *SaTT12* and *SaABI3* genes.

plaa044_suppl_Supplementary_Figure_S1

plaa044_suppl_Supplementary_Tables

plaa044_suppl_Supplementary_Data

## Data Availability

The raw data used in this study are also available as **Supporting Information**.
