# Supplementary material for: Effects of genetic and environmental factors on variations of seed heteromorphism in Suaeda aralocaspica
Source: AoB Plants. 2020 Aug 24;12(5):plaa044. doi: 10.1093/aobpla/plaa044 (PMC7546916; doi:10.1093/aobpla/plaa044)

**Figure S1.** Seed viability and germination characteristics in *S. aralocaspica*. A-J: morphology of the embryo stained by TTC after different treatments; A-E: large black seeds; F-J: small black seeds; A, F: embryos before TTC staining; B, G: distilled-water treatment; C, H: NaCl treatment; D, I: PEG treatment; E, J: 4°C treatment, scale bar=1 mm. K: viability of large black and small black seeds; L: germination of three types of seeds under different treatments. Control: distilled water; NaCl: 300 mmol·L^-1^; PEG: 20%; 4°C: germination under 4°C for 15 d. Different lowercase letters in L indicate signiﬁcant differences (*P*<0.05) under the same treatment; *, **, ***: indicate significance existing in the same seed type at 0.05, 0.01, 0.001 level. Values are means ± SE of (1) four replicates with 30 seeds of each in germination; (2) three replicates with 15 seeds of each from the ungerminated black seeds stained and calculated.


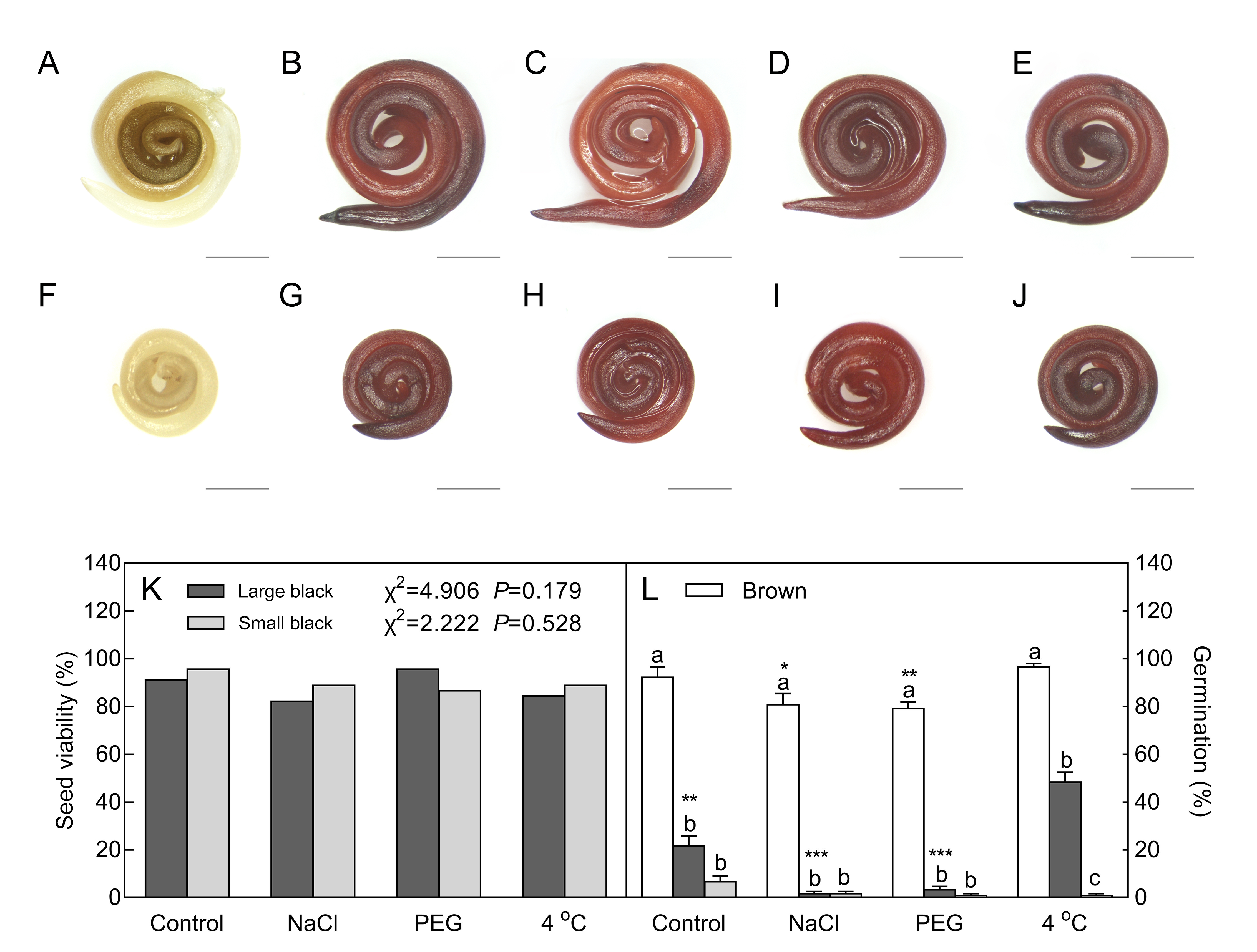

Supplement: plaa044_suppl_Supplementary_Figure_S1 [file plaa044_suppl_supplementary_figure_s1.docx]
