# Supplementary material for: Effects of genetic and environmental factors on variations of seed heteromorphism in Suaeda aralocaspica
Source: AoB Plants. 2020 Aug 24;12(5):plaa044. doi: 10.1093/aobpla/plaa044 (PMC7546916; doi:10.1093/aobpla/plaa044)
Supplement: plaa044_suppl_Supplementary_Tables [file plaa044_suppl_supplementary_tables.docx]

Table S1 The components and the physical and chemical properties of the soil in the natural habitat of *Suaeda aralocaspica*

| Depth  (cm) | Organic subs.^§^  (g/kg) | [Conduct.](app:ds:conductance)^¶^  (ms/cm) | Total salt  (mg/g) | pH | Cl^-^  (mg/g) | SO_4_^2-^  (mg/g) | Ca^2+^  (mg/g) | K^+^  (mg/g) | Mg^2+^  (mg/g) | Na^+^  (mg/g) | CO_3_^2-^  (mg/g) | HCO_3_^-^ (mg/g) |
| --- | --- | --- | --- | --- | --- | --- | --- | --- | --- | --- | --- | --- |
| 0 | 16.08 | 24.40 | 132.00 | 8.37 | 14.67 | 73.10 | 2.19 | 0.12 | 0.07 | 31.85 | 0.05 | 0.12 |
| 5 | 19.90 | 20.20 | 97.45 | 8.67 | 10.26 | 57.61 | 2.19 | 0.15 | 0.07 | 25.00 | 0.09 | 0.09 |
| 10 | 19.72 | 11.69 | 55.75 | 8.80 | 9.24 | 29.28 | 1.35 | 0.18 | 0.05 | 12.24 | 0.14 | 0.05 |

^§^ subs., substance; ^¶^ [conduct.](app:ds:conductance), conductance

Table S2 Quantitative PCR (qRCR) primers of *SaANS, SaBAN, SaTT12,* and *SaABI3* genes

| Gene | Full name | Primer sequence 5’-3’ | |
| --- | --- | --- | --- |
|  |  | Forward | Reverse |
| *SaANS* | Anthocyanin synthase | AGCCTTCCTTCTTCCAACCC | TACACATCCGCCGTTTCAG |
| *SaBAN* | BANYULS (anthocyanidin reductase) | GCCGTTTGTTCTGCTAGTGTC | CGAAGGCAAAGTCTGTGAAAC |
| *SaTT12* | Transparent testa 12 | GCCAGATTCCTGTGAAAGC | TTGACTATGGACTTATCGGAGC |
| *SaABI3* | Abscisic acid insensitive 3 | GACGCTATAGCGCATGTTCC | GTGATGTTGGTAATCTTGGCAG |
| *Saactin* |  | CCAAAGGCCAACAGAGAGAAGAT | TGAGACA CACCATCACCAGAAT |
